# Improving the Solubility and Bioavailability of Progesterone Cocrystals with Selected Carboxylic Acids

**DOI:** 10.3390/pharmaceutics16060816

**Published:** 2024-06-16

**Authors:** Jing Xiong, Dezhong Xu, Hui Zhang, Yan Shi, Xiangxiang Wu, Sicen Wang

**Affiliations:** 1 School of Pharmacy, Health Science Center, Xi’an Jiaotong University, Xi’an 710061, China; xiongjing@nifdc.org.cn; 2 Institute for Chemical Drug Control, National Institutes for Food and Drug Control, Beijing 102629, China; 3 Key Laboratory of Radiopharmaceuticals of Ministry of Education, College of Chemistry, Beijing Normal University, Beijing 100875, China; 202231150023@mail.bnu.edu.cn; 4 Pharmacy College, Henan University of Chinese Medicine, Zhengzhou 450046, China; zz__28@163.com; 5 Institute for Chinese Traditional Medicine Control, National Institutes for Food and Drug Control, Beijing 102629, China; san0373@163.com

**Keywords:** pharmaceutical cocrystal, crystal engineering, solubility, permeability, oral bioavailability, progesterone

## Abstract

Progesterone (PROG) is a natural steroid hormone with low solubility and high permeability that belongs to biopharmaceutics classification system class II. In this study, novel pharmaceutical cocrystals of PROG were successfully prepared by solvent evaporation or a liquid-assisted grinding process aimed at enhancing its solubility and bioavailability. The cocrystal formers selected based on crystal engineering principles were carboxylic acids, namely, 4-formylbenzeneboronic acid (BBA), isophthalic acid (IPA), and 3-nitrophthalic acid (NPA). The cocrystal structures were characterized using multiple techniques. Single-crystal X-ray diffraction results showed that the carbonyl group, acting as a hydrogen bond acceptor, was pivotal in the cocrystal network formation, with C–H···O interactions further stabilizing the crystals. The cocrystals exhibited improved solubility and dissolution profiles in vitro, with no significant changes in hygroscopicity. The parallel artificial membrane permeability assay (PAMPA) models indicated that the cocrystals retained PROG’s high permeability. Pharmacokinetic studies in Sprague–Dawley rats revealed that all cocrystals increased PROG exposure, with AUC_(0~∞)_ values for PROG–BBA, PROG–IPA, and PROG–NPA being 742.59, 1201.72 and 442.67 h·ng·mL^−1^, respectively. These values are substantially higher compared to free PROG, which had an AUC_(0~∞)_ of 301.48 h·ng·mL^−1^. Notably, PROG–IPA provided the highest AUC improvement, indicating a significant enhancement in bioavailability. Collectively, the study concludes that the cocrystal approach is a valuable strategy for optimizing the physicochemical properties and oral bioavailability of PROG, with potential implications for the development of other poor water-soluble drugs.

## 1. Introduction

Progesterone (PROG) is a natural steroid hormone that is essential in menstrual cycle, pregnancy, reproduction, and fetal development; low PROG levels in the body can cause side effects [1]. PROG is a Biopharmaceutics Classification System (BCS) class II drug with low solubility and high permeability [2,3]. The water solubility of PROG at room temperature was determined to be only 5.46 mg/L. So PROG exhibits low oral bioavailability and poor absorption in vivo [4]. In clinical practice, PROG is generally administered via intramuscular injection; however, intramuscular injection is inconvenient for patients because it often requires long-term administration throughout treatment. Therefore, developing a suitable method to improve the oral bioavailability of PROG is important.

In recent years, various methods, including nanostructures [5], polymer delivery systems [6], and prodrug design [7], have been developed to improve drug dissolution and bioavailability. These methods offer unique characteristics that are not always achievable with simpler methods, and the costs are high. Therefore, exploring a low-cost strategy to boost the water solubility of PROG while preserving its high membrane permeability is crucial in increasing its bioavailability. Cocrystals are multi-component crystalline materials composed of two or more constituents bound by abundant noncovalent interactions, such as hydrogen bonding, π–π stacking, and Van der Waals interactions [8]. In recent years, pharmaceutical cocrystals have received extensive attention as unique solid forms [9]. Compared with other solid drug forms, pharmaceutical cocrystals prepared by rational design can improve the physical properties of active pharmaceutical ingredients (APIs) without changing their structure and exhibit controlled thermochemical stability and economic accessibility [10].

Weak interactions are the main driving forces for cocrystal formation. Following crystal engineering principles, APIs and cocrystal formers (CCFs) will increase the likelihood of generating cocrystal networks. Specifically, the hydrogen bonding force between an API and CCF is important and widely used in cocrystal design because of its directionality, strength, and commonality in organic materials [11]. API- or CCF-containing amino, carboxyl, and carbonyl groups frequently appear in pharmaceutical cocrystals [12] and can provide abundant hydrogen bond donors and acceptors. In recent years, various inorganic and organic acids, such as oxalic acid, malonic acid, gentisic acid, maleic acid, p-aminobenzoic acid, and adipic acid, have been selected as CCFs to form various cocrystals [13,14]. For example, the physicochemical properties of glibenclamide can be improved by enhancing its solubility [15].

PROG is an ideal API for cocrystals because its structure contains a steroid backbone and terminal carbonyl groups, which provide the possibility of forming a complex intermolecular network [16]. Several PROG cocrystals, including alcohols, phenols, aromatic carboxylic acids, and theophylline, have been reported in recent years [17,18]. However, most studies have focused on intermolecular forces, and in vivo or in vitro behaviors have rarely been reported. In our previous work, we selected 2-chloro-4-nitroaniline (CNA), 2,5-dihydroxybenzoic acid, and 4,4′-phenol as CCFs to form cocrystals with PROG [19]. Compared with free PROG, all cocrystals, especially PROG–CNA, showed better solubility and dissolution rate. Building on these findings, we expand the scope of CCFs to include small-molecule acids, which may offer additional advantages in terms of forming stable cocrystals with improved pharmaceutical properties.

**Scheme 1:**
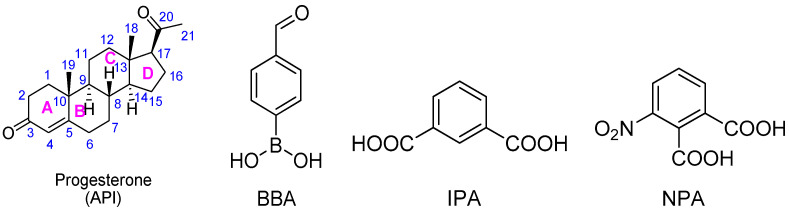
Structures of PROG and CCFs.

In the present study, we screened three small-molecule acids, 4-formylbenzeneboronic acid (BBA), isophthalic acid (IPA), and 3-nitrophthalic acid (NPA), as CCFs to construct PROG cocrystals according to the crystal engineering strategy. The structures of PROG and CCFs are depicted in Scheme 1. The formation, structure, and physicochemical properties of the cocrystals were comprehensively characterized using single-crystal X-ray diffraction spectroscopy (SCXRD), powder X-ray diffraction spectroscopy (PXRD), fourier transform infrared spectroscopy (FTIR), differential scanning calorimetry (DSC), and dynamic vapor sorption (DVS). Solubility and dissolution behaviors were explored using previously described methods [19]. 

Increased solubility does not necessarily mean increased absorption, sometimes even the opposite [20]. The solubility–permeability interplay is complicated by many physicochemical elements, physiological characteristics, and dose form-related factors [21]. Therefore, changes in the permeability of the cocrystals must also be explored. In recent years, parallel artificial membrane permeability assay (PAMPA) models have been widely used in drug discovery to determine the absorption potential of compounds [22]. In the present study, we also performed a *μ*Flux test of PAMPA models to evaluate the in vitro permeability of the cocrystals. Furthermore, the pharmacokinetics of the cocrystals were evaluated using Sprague–Dawley (SD) rats. This study provides compelling evidence that the pharmaceutical cocrystal method can be successfully employed on poorly water-soluble PROG to improve its in vivo bioavailability.

## 2. Materials and Methods

### 2.1. Materials

Methanol and ethanol were of analytical reagent grade. PROG (99% purity) was purchased from TCI Chemical Industry Development Co., Ltd. (Shanghai, China). BBA (97% purity) was purchased from Alfa Aesar Co., Ltd. (Shanghai, China). IPA (99%) and NPA (96%) were obtained from Sinopharm Chemical Reagent Co., Ltd. (Shanghai, China). Distilled water was obtained using a Milli-Q^®^ Integral 5 water-purification system (Merck, Darmstadt, Germany). All the materials were used in these experiments without additional purification. Male SD rats (180–220 g) were purchased from Jinan Pengyue Experimental Animal Breeding Co., Ltd. (Production license No.: SCXK (Yu) 2021-0015, Zhengzhou, China). All animal experiments were performed following a protocol approved by the Animal Care and Use Committee of Henan University of Chinese Medicine.

### 2.2. Preparation of PROG Cocrystals

PROG–BBA, PROG–IPA, and PROG–NPA were prepared by liquid-assisted grinding. The experiments were performed by addition of an equimolar of PROG (157.2 mg, 0.5 mmol) and corresponding CCF into a 25 mL stainless steel grinding jar. Two drops of methanol/ethanol/water (1:1:1, *v*/*v*/*v*) for PROG–BBA or PROG–IPA and two drops of ethanol/water (1:1, *v*/*v*) for PROG–NPA were added for facilitating the reaction. The mixture was then ground at a frequency of 20 Hz for 30 min using a stainless steel grinding ball.

Single cocrystals were obtained via a slow-evaporation technique by dissolving equimolar amounts of PROG (314.5 mg, 1 mmol) and the corresponding CCF in an appropriate solvent. Suitable single crystals of PROG–IPA and PROG–NPA were obtained from ethanol/water (1:1, *v*/*v*) after a period of 5 and 12 days, respectively. However, it was confirmed that the stoichiometric ratio of PROG–IPA in the resulting crystal was 2:1. Additionally, PROG–BBA crystals were grown in methanol/ethanol/water (1:1:1, *v*/*v*/*v*) over 7 days.

### 2.3. Powder X-ray Diffraction (PXRD)

A Rigaku Smartlab 9 kw X-ray diffractometer (Rigaku Corp., Akishima-shi, Tokyo, Japan) was utilized for PXRD measurements. The instrument was equipped with a CuKα radiation source that emits radiation with a characteristic wavelength of 1.5418 Å. The device was operated at a voltage of 45 kV and a current of 200 mA. The standard range for the scan was set from 5° to 40°, which is a typical range used to capture the diffraction pattern of crystalline materials. The scanning speed was 40°/min and the step size was adjusted to 0.01°.

### 2.4. Single-Crystal X-ray Diffraction (SCXRD)

Single-crystal X-ray data were obtained using a standard procedure on a Bruker APEX II charge-coupled device diffractometer at 25 °C. The structures were determined by employing direct approaches and full matrix least-square methods based on *F*^2^. Data collection and structure refinement or graphics were performed using the SHELXTL package (Version: 2014). Platon software (Version: 261023) was used to calculate the weak interaction.

### 2.5. IR Spectroscopy

IR spectroscopy was performed on a Spectrum 100 FTIR Spectrometer (PerkinElmer, Waltham, MA, USA). The powdered samples and potassium bromide (approximately 1:200 mg) were ground using an agate mortar. For each sample, 32 scans with the resolutions of 4 cm^−1^ were recorded. And the spectrometer scan range was 4000–400 cm^−1^.

### 2.6. Differential Scanning Calorimetry (DSC)

The thermal behavior of the samples was investigated using a Mettler Toledo DSC 822^e^ (Mettler-Toledo Instruments Co., Ltd., Zurich, Switzerland). Each sample (1–5 mg) was placed into an aluminum crucible and subjected to a heating rate of 10 °C/min. The temperature was ramped from an initial 30 °C up to a final temperature of 200 °C, 300 °C, or 375 °C, depending on the observed melting endotherm of the samples. This process was carried out under a nitrogen flow of 50 mL/min. For comparison, an empty aluminum crucible was used as a reference.

### 2.7. Dynamic Vapor Sorption (DVS)

DVS studies were performed using an DVS Advantage apparatus (Surface Measurement Systems Ltd., London, UK). The samples were loaded into the aluminum pan, with the respective masses of PROG–BBA, PROG–IPA, PROG–NPA and PROG being 39.4 mg, 38.7 mg, 32.0 mg and 39.9 mg. The analysis was performed at a constant temperature of 25 °C. A nitrogen flow of 200 mL/min was used to equilibrate the samples, which served to remove any residual moisture and establish a baseline dry mass. The RH was set to change from 20% to 80% in 10% steps and then decrease to 0% in a similar stepwise fashion. At every stage, the RH was kept constant for 1 h, with the exception of RH80% for 3 h. The resulting sorption and desorption data were recorded and analyzed using the DVS Advanced Analysis Suite software (Version: 3.6).

### 2.8. In Vitro Dissolution

Dissolution tests were conducted using an RC806D dissolution equipped with a USP apparatus II (paddle-type) (Tianjin Tianda Tianfa Technology Co., Ltd., Tianjin, China). The tests utilized simulated gastric fluid at pH 1.2 and distilled water as the dissolution media. PROG (30 mg) and its cocrystals (containing 30 mg of PROG) were added to 100 mL of the respective dissolution media. The resulting slurries were stirred at a controlled temperature of 37.0 ± 0.5 °C and a constant speed of 100 rpm. At predetermined time intervals of 5, 10, 15, 20, 30, 40, 60, 90, and 120 min, samples were taken from the dissolution media. The dissolution medium was replenished at 37 °C with an equal volume of fresh medium. These samples were then filtered through a 0.22 μm membrane filter labeled as MCM and subsequently analyzed by high-performance liquid chromatography (HPLC) to determine the concentration of PROG present. The HPLC system utilized Empower 3 software (Version: FR4 SR3, Waters Technology, Milford, MA, USA) and was equipped with a photo-diode array (PDA) detector. The mobile phase was a mixture of methanol, acetonitrile, and water in the ratio of 25:35:40. This phase was degassed and delivered at a flow rate of 1.0 mL/min through a reversed-phase column, which was a BDS hypersil C8 column with 250 mm × 4.6 mm (i.d.), featuring an average particle size of 5 µm. The eluent was monitored at a wavelength of 241 nm for detection purposes [19].

### 2.9. μ*Flux* Measurements

In this work, the permeation rate and effective permeability of PROG and its cocrystals were determined using a Pion µFlux profiler (Pion Inc., Billerica, MA, USA). The profiler consisted of donor and acceptor chambers separated by a vertically positioned biomimetic membrane. The membrane was impregnated with a gastrointestinal lipid solution (GIT Lipid) to form a PAMPA membrane as a permeation barrier. PROG and its cocrystals (equivalent to PROG, 0.5–3 mg) were placed in the donor chambers simulating the gastrointestinal tract, where 20 mL of pH 6.8 phosphate-buffered saline (PBS) was added. The acceptor chambers simulating the blood circulation and 20 mL of acceptor sink buffer (ASB, Pion Inc., Billerica, MA, USA) were added. ASB is a HEPES buffer (pH 7.4) containing surfactants micelles to maintain sink conditions during the experiments. PBS and ASB were both maintained at 37 °C. Prior to the measurement, the membrane was formed by placing 25 μL of GIT Lipid (Pion Inc., Billerica, MA, USA) on the filter supported hydrophobic material (polyvinylidene fluoride, 1.54 cm^2^ open area, 0.45 μm pore size). All experiments were conducted at 37 °C with stirring at 200 rpm using cross-bar magnets. The UV signal of PROG in the acceptor chambers was constantly monitored in situ using the integrated fiber optic UV-vis probes at the ultraviolet wavelength range of 250–350 nm, and the lengths of the probe tips were 5 mm in pH 6.8 PBS and 20 mm in ASB. The acquisition time was set at 4 h, and the sampling interval was set at 60 s. According to the concentration–time profiles obtained in the acceptor chambers, the flux (*J*) and effective permeability (*P*e) were calculated using the following equations:

(1) $$J=\frac{\mathrm{dc}}{\mathrm{dt}}\times \frac{V}{A}$$

(2) $$Pe=\frac{\mathrm{dc}}{\mathrm{dt}}\times \frac{V}{A\times Ct\times 60}$$

where the permeation rate flux (*J*), is the amount of PROG transfer through the membrane (μg·min^−1^·cm^−2^); *P*e is the effective permeability (cm·s^−1^); $\frac{\mathrm{dc}}{\mathrm{dt}}$ is the slope of the concentration–time profile of PROG in the acceptor chamber (μg·mL^−1^·min^−1^); *A* is the area of the membrane (cm^2^); *V* is the volume of the buffer in the acceptor chamber (mL); and *C*t is the initial concentration of drug in the donor chamber.

### 2.10. Pharmacokinetic Study

The pharmacokinetics of PROG and its cocrystals were evaluated in male SD rats. The rats were kept under standard housing conditions (in an environment with a light–dark cycle at 25 ± 1 °C), meanwhile water and food were provided. Before the experiment, the rats were fasted for 12 h and allowed to drink freely. 

Sixteen rats were randomly divided into four groups (*n* = 4/group) and orally administered PROG (50 mg/kg) or PROG cocrystals (50 mg/kg PROG equivalent). After oral administration, blood samples of 500 μL were collected from the small saphenous vein on the lateral hindlimb at the following time intervals (0.01, 0.25, 0.5, 1, 2, 4, 6, 8, 12, and 24 h). Subsequently, the samples were collected in 1.5 mL test tubes containing heparin sodium to prevent blood clotting. Then, the blood samples were centrifuged at 3000 rpm for 10 min, and the resultant supernatant was stored at −80 °C.

Following a series of preliminary studies, the following plasma processing procedure was employed. First, each plasma sample (100 µL) was extracted with 400 µL of methanol, and then the internal standard of megestrol acetate (10 µL, 1 µg/mL in methanol) was added. The mixture was vortexed for 3 min and allowed to stand for 10 min. The suspension was centrifuged at 12,000 rpm for 10 min. Then, 380 μL of the upper solvent was collected into a centrifuge tube. After most of the solvent was removed, the suspension was redissolved in 100 µL of the methanol/water solution (70:30, *v*/*v*). After vortex and centrifugation, the supernatant was used for UPLC-MS analysis on a Thermo Scientific TSQ Altis Triple Quadrupole Mass Spectrometer (Thermo Fisher Scientific Inc., Waltham, MA, USA) with a Hypersil GOLD™ C18 (100 mm × 2.1 mm, 1.8 μm). The column was eluted with the mobile phase at 0.2 mL/min (0.1% formic acid-water (A) and acetonitrile (B), gradient elute). Column temperature: 35 °C, injection volume: 3 μL. Quantitative analyses of the standard curves, precision, stability, recovery, and matrix effects were conducted before the formal test [23].

## 3. Results

### 3.1. Single-Crystal X-Ray Diffraction

The crystal structure parameters are listed in Table 1. The multiple hydrogen bonds formed between PROG and the CCFs in the single-crystal structure of the cocrystals are shown in Table 2. The carbonyl group, as the hydrogen bond acceptor, played a crucial role in cocrystal network formation, and the weaker C–H···O bond further enhanced crystal stability. 

**Figure 1:**
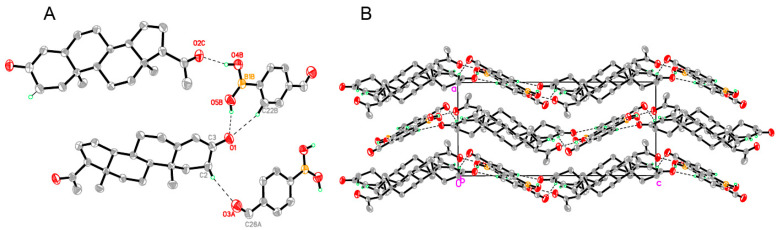
(**A**) Hydrogen-bond pattern of PROG–BBA of O–H···O hydrogen bonds (dashed lines) and C–H···O weak interaction. (**B**) Projection of PROG–BBA packing diagram along the *b* axis. (The ellipsoids are drawn with 30% probability, and all hydrogen bonds are represented by dotted lines. All H atoms that were not involved in hydrogen bonding were omitted for clarity. This was also performed in the schematics below. Symmetry codes: A: 1 − x, 1/2 + y, 3/2 − z; B: x, 1 + y, z; C: 1 − x, 1/2 + y, 1/2 − z).

The expected 1:1 molecular stoichiometry of PROG–BBA was determined from its crystal structure (Figure 1A). The crystal belongs to the orthorhombic system and has a chiral space group of *P*2_1_2_1_2_1_. The structure of PROG and BBA is stabilized by abundant O–H···O hydrogen bonding and weak C–H···O interactions (Table 2), with O(5B)···O(1), C(22B)···O(1), O(4B)···O(2C), and C(2)···O(3A) distances of 2.801 (3), 3.456 (3), 2.802 (3), and 3.417 (4) Å, respectively. The connection mode involved the closed cavity of the $R_{2}^{1}(7)$ ring motif. PROG and the adjacent BBA are linked by weak C(2)···O(3A) interactions (Figure 1A). The layers extend along the (100) plane (Figure 1B), with an inter-distance of approximately 4.5 Å.

In the cocrystal, the configuration of BBA molecules has undergone slight changes. The dihedral angle between the benzene ring and the B(OH)_2_ group was 9.9° in PROG–BBA, which is lower than the 20.6° of the BBA itself [24]. This change may have been caused by the formation of C–H···O weak interaction and O–H···O hydrogen bonds between BBA and PROG. Similar to the structure of the BBA molecule alone, one hydroxyl H atom of the B(OH)_2_ group was syn with the phenyl group, whereas the other was anti, as is typical of phenylboronic acids.

PROG–IPA possessed a monoclinic crystal system, with the space group *C*_2_. The benzoic acid family possesses robust hydrogen bonding capacity and is widely used in the formation of organic cocrystals [25,26]. Most of the hydrogen bonding modes involved the combination of strong (e.g., O–H···O, O–H···N or N–H···O) and weak (e.g., C–H···O) interactions [27]. For example, the cocrystal of IPA and 1,4-bis[(imidazol-1-yl)methyl] benzene formed the intended infinite one-dimensional chain via COOH···Im hydrogen bonds, accompanied by a C–H···O from Im and the carboxyl group^44^.

**Figure 2:**
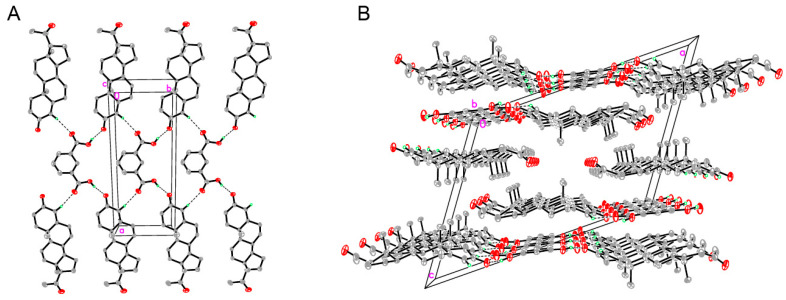
(**A**) Weak interaction pattern of PROG–IPA. Sheet structure along the (001) plane is sustained by C–H···O and O–H···O interactions. (**B**) Projection of the packing diagram of PROG–IPA along the *b* axis.

As shown in Figure 2, PROG–IPA contained PROG, IPA, and water at a stoichiometry ratio of 2:1:1. The PROG and IPA molecules were bound by multiple hydrogen bonds between the keto carbonyl of PROG and the carboxyl group of IPA. The sheet structure along the (001) plane was sustained by C(4)–H(4A)···O(3) and O(4)–H(4)···O(1) interactions, in an $R_{4}^{4}(24)$ ring motif and $C_{2}^{2}(9)$ chain motif, with C(4)···O(3) and O(4)···O(1) distances of 3.328 (13) Å and 2.613 (10) Å, respectively. The rings were aligned along the *b* axis, and 2D layer modes were formed. The sheet structure was similar to that of the isophthalic acid:bipy-pr cocrystal [27]. Water molecules did not participate in any interactions and were packed into the sheet structure.

**Figure 3:**
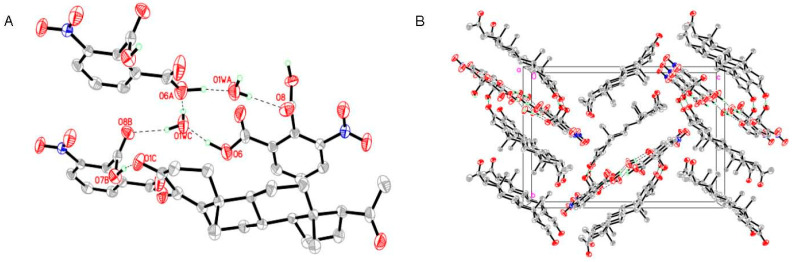
(**A**) Hydrogen bond PROG–NPA patterns. (**B**) Packing diagram of a PROG–NPA cocrystal along the *a* axis. (Symmetry codes: A: 1 − x, 3/2 − y, 1 − z; B: 2 − x, 3/2 − y, 1 − z; C: 3/2 − x, y, z).

The PROG–NPA crystal exhibited an orthorhombic crystal system with the chiral space group *P*2_1_2_1_2_1_. The asymmetric unit of the PROG–NPA crystal comprised equal amounts of PROG, NPA, and water (Figure 3). The existence of water molecules in PROG–NPA caused the formation of a network that is significantly different from that in PROG–IPA or NPA alone [28]. In PROG–IPA, water molecules did not participate in any interactions, whereas in PROG–NPA, they played a significant role in network formation. Specifically, two water molecules linked the adjacent NPA molecules via O–H···O hydrogen bonds in the $R_{4}^{4}(13)$ ring motif. The enclosed ring further linked another NPA molecule through O–H···O hydrogen bonds, with the O(8)···O(1W) distance of 2.851 (1) Å. The NPA molecule bound to the adjacent PROG molecule through O–H···O hydrogen bonds, with the O(7)···O(1) distance of 2.591 (3) Å, to form the three-dimensional structure of PROG–NPA (Figure 3B).

### 3.2. Powder X-ray Diffraction

**Figure 4:**
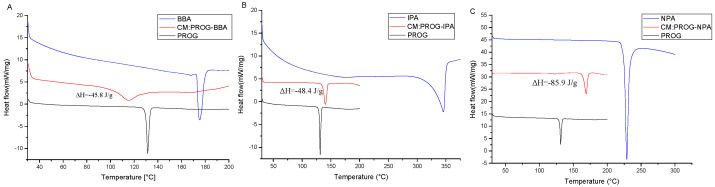
Combined DSC plots of (**A**) BBA, PROG–BBA, PROG; (**B**) IPA, PROG–IPA, PROG; and (**C**) NPA, PROG–NPA, PROG.

PXRD is a powerful technique for determining cocrystal formation, and the appearance of new derivative peaks often implies the generation of new phases. The PXRD patterns of the three cocrystals and free PROG are shown in Figure S1 (Supporting Information). The PXRD patterns of PROG–BBA, PROG–IPA, and PROG–NPA differed from those of PROG and its coformers, indicating the formation of new crystal structures. Moreover, the patterns of the cocrystals, except for PROG–NPA, were consistent with the simulated diffraction line pattern from the X-ray crystal structure. The difference may be due to the occurrence of a crystal transformation during the grinding of the sample, and this point was supported by DSC results of PROG–NPA in Figure 4. Further experiments on the polymorph of PROG–NPA will be carried out. Specifically, the characteristic 2θ peaks of PROG–BBA acquired by PXRD were located at 9.04°, 13.44°, 16.48°, 20.70°, and 23.48°. The characteristic 2θ peaks of PROG–IPA appeared at 6.56°, 13.14°, 16.17°, 19.76°, 22.20°, 26.45°, and 28.55°. The characteristic 2θ peaks of PROG–NPA were 9.08°, 13.01°, 13.39°, 13.78°, 15.88°, 18.78°, 19.29°, 27.33°, and 29.79°. These results further verify the cocrystallization of PROG with the coformers.

### 3.3. IR Spectroscopy

IR spectroscopy can provide information about noncovalent bonds through characteristic peak shifts. The IR spectra of each cocrystal were obviously different from those of PROG and CCFs, as shown in Figure S2 (Supporting Information). The carbonyl groups in both PROG and CCFs exhibited shifts in their IR absorption bands within the range of 1635 to 1701 cm^−1^, which can be attributed to the hydrogen bonding interaction between the carbonyl group and a nearby hydroxyl group, denoted as -C=O···H–O. The stretching frequencies of 3406 cm^−1^ in PROG–BBA and 3569 cm^−1^ in PROG–IPA were possibly caused by -C···H–O weak hydrogen bonds. These results are consistent with those of the single-crystal structure.

### 3.4. Differential Scanning Calorimetry

The thermal behavior of the cocrystals of PROG and the CCFs was characterized by DSC, and the DSC plots are shown in Figure 4. PROG–BBA showed an endothermic peak at 114.7 °C (ΔH = −45.8 J/g), which was lower than that of PROG and CCF (130.9 °C and 174.1 °C). The endothermic peaks of PROG–IPA and PROG–NPA appeared at 140.1 °C (ΔH = −48.4 J/g) and 169.0 °C (ΔH = −85.9 J/g), respectively, which were between PROG and their corresponding CCFs (IPA: 344.6 °C; NPA: 225.5 °C). PROG–NPA cocrystal specially showed a small endothermic peak at 122.5 °C before melting, which indicated that a phase transition or polymorphism may have occurred during the heating process. The outcomes comply with the relevant rules; that is, the endothermic peaks of the cocrystals were always between or below those of the API or CCF [10].

### 3.5. Dynamic Vapor Sorption

The hygroscopicity of PROG and its cocrystals was determined using DVS, and the results are shown in Figure S3 (Supporting Information). The relative mass change of PROG was less than 0.2% when exposed to a certain humidity during the test, indicating that PROG was not hygroscopic. The relative mass change of the cocrystals was less than 0.2% under the same conditions, suggesting that the cocrystals maintained the nonhygroscopicity of PROG. Notably, this characteristic is beneficial for the stability of the PROG cocrystals.

### 3.6. In Vitro Dissolution

**Figure 5:**
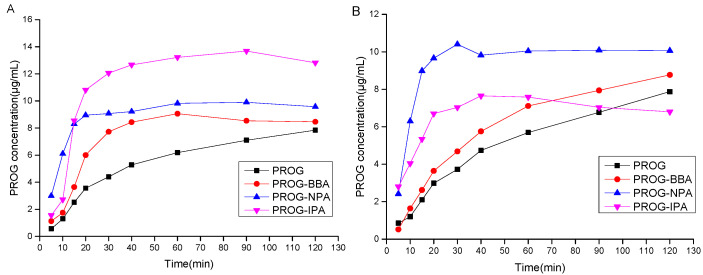
Dissolution curves of PROG and its cocrystals at 37 ± 0.5 °C. (**A**) Water. (**B**) pH 1.2 aqueous solution.

Cocrystal formation improved the solubility and dissolution rate of PROG. As shown in Figure 5A, in water medium at 37 ± 0.5 °C, PROG–IPA, PROG–BBA, and PROG–NPA reached *C*_max_ of 13.68, 9.06, and 9.90 μg/mL, respectively, which were 1.7, 1.2, and 1.3 times that of PROG (7.84 μg/mL). Moreover, the cocrystals exhibited 2–4 times faster dissolution rates than PROG during the first 30 min. Among the cocrystals, PROG–IPA exhibited the highest *C*_max_ and the fastest dissolution rate. However, the difference was reduced in pH 1.2 aqueous solution at 37 ± 0.5 °C. The solubility and dissolution rate of PROG–NPA obviously improved, whereas those of PROG–IPA increased in the first 40 min and then declined slowly. PROG–BBA showed a similar trend to that of PROG, and a slight improvement was observed. Among the cocrystals, only PROG–IPA exhibited an obvious reduction in solubility with decreasing pH indicating that H^+^ disrupted the hydrogen bond interaction between PROG and IPA and thus decreased in the solubility of PROG. 

### 3.7. In Vitro Permeation

**Figure 6:**
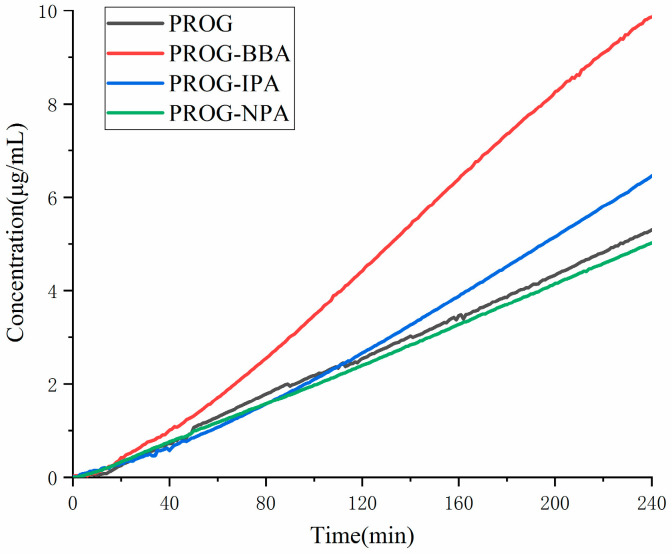
Permeation rate curves of PROG and its cocrystals in pH 6.8 PBS.

The permeation rate and effective permeability of the PROG monomer and cocrystals were investigated using *μ*Flux in PBS (Figure 6). The drug was gradually transferred from the donor chamber to the acceptor chamber through the PAMPA membrane. According to Equation (1), the *Flux*(*J*) values of PROG–IPA, PROG–BBA, and PROG–NPA were 0.36, 0.62, and 0.28 μg·min^−1^·cm^−2^, respectively, all of which were 1.1, 1.9, and 0.9 times higher than the 0.32 μg·min^−1^·cm^−2^ of PROG. Among the cocrystals, PROG–BBA showed the most marked increase.

In general, medications with higher permeability than metoprolol are regarded as permeable medications, whereas those with lower permeability are regarded as low-permeability drugs. In the present study, the effective permeability (*P*e) of PROG was calculated using Equation (2) to be 1.11 × 10^−3^ cm·s^−1^. The *P*e values of PROG–BBA, PROG–IPA, and PROG–NPA were 8.87 × 10^−4^, 9.38 × 10^−4^, and 1.46 × 10^−3^ cm·s^−1^, respectively, all of which were higher than the 1.20 × 10^−4^ cm·s^−1^ of metoprolol. These results indicated that the cocrystal formation maintained the high permeability properties of PROG, allowing PROG to exert its effects in vivo.

### 3.8. Pharmacokinetic Study

The above study confirmed that the cocrystal formation increased solubility and dissolution rate to varying degrees. All cocrystals maintained a permeability that is similar to or higher than that of PROG. In general, these properties are beneficial for improving drug bioavailability in vivo. Hence, we performed pharmacokinetic tests in rats to investigate their in vivo relevance. The quantitative analysis indicated that the experimental scheme met the requirements for pharmacokinetic studies. In the rat plasma samples, the calibration curves were linear over the concentration range of 0.4–800 ng/mL, with a correlation coefficient (*R*^2^) of 0.9994. The intra- and inter-day precision values (RSD) of the PROG samples with low (4 ng/mL), medium (40 ng/mL), and high concentrations (400 ng/mL) were less than 10%, with corresponding accuracy values of 103.57 ± 8.8%, 103.41 ± 3.24%, and 96.71 ± 0.86%; extraction recovery rates of 99.03 ± 2.68%, 86.30 ± 1.54%, and 92.64 ± 4.40%; and matrix effect values of 98.59 ± 3.04%, 98.87 ± 1.34%, and 98.97 ± 1.15%.

**Figure 7:**
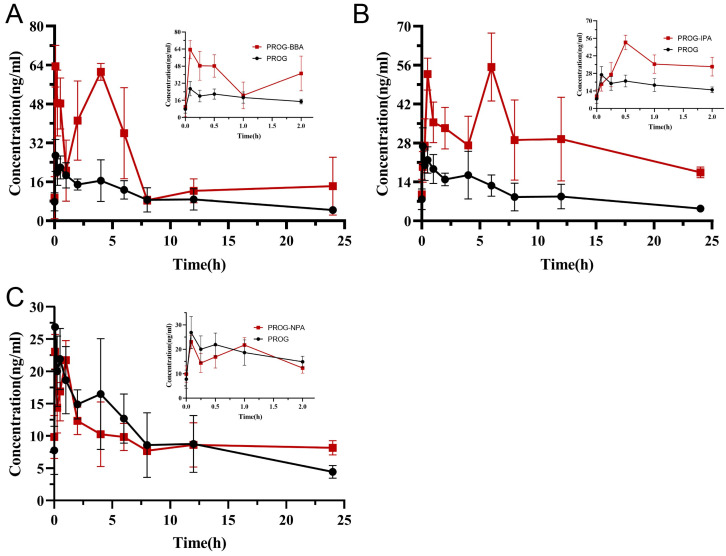
Mean plasma concentration versus time curves of PROG after oral administration of free PROG (black circles) and its cocrystals (red squares, (**A**): PROG–BBA, (**B**): PROG–IPA, (**C**): PROG–NPA) in SD rats. Each point represents the mean ± SD (*n* = 4).

The pharmacokinetic data of all samples were analyzed using the PK Solver 2.0 software. A noncompartmental model was used to fit the blood concentration data. The calculated pharmacokinetic parameters are presented in Table S1. The half-life (*t*_1/2_) values of all cocrystals, except for PROG–BBA, were almost twice that of free PROG indicating that a slower elimination rate for the cocrystals. Moreover, the cocrystals increased the exposure of PROG, with AUC_(0~∞)_ values 1–3 times that of free PROG. The rats that received PROG–IPA had the highest AUC value, with approximately 3–4-fold improvements in AUC_0–24 h_ and AUC_0–∞_. The mean plasma concentration versus time curves of PROG in the different groups are shown in Figure 7. These results revealed that the cocrystal strategy effectively improved the bioavailability of PROG.

## 4. Discussion

The cocrystal’s hydrogen bonding network, particularly the carbonyl group of PROG acting as a hydrogen bond acceptor, is crucial for the formation and stability of the cocrystal lattice. This network can significantly affect the solubility, dissolution rate and permeability, which are key parameters for oral bioavailability, and should be of particular concern in the cocrystal strategies. Treating permeability and solubility independently may not be sufficient because they are closely connected [20]. 

In the present study, the following trends were observed: in vitro dissolution (pH 1.2, 37 °C), PROG–NPA > PROG–IPA > PROG–BBA > PROG; in vitro permeation (*μ*Flux test), PROG–BBA > PROG–IPA > PROG > PROG–NPA; pharmacokinetics study (AUC): PROG–IPA > PROG–BBA > PROG–NPA > PROG. Almost all the cocrystals showed higher solubility and bioavailability than PROG, and abundant hydrogen bonding network may be favorable to the process. 

Moreover, for cocrystals containing only a single active pharmaceutical ingredient, adding highly soluble CCFs often enhances the solubility of the material [29,30]. Here, the improvement of solubility of the PROG may be attributed to the property of CCF candidates, since all the CCFs in the paper have more than 20 times higher water solubility than PROG. Despite the preferable stability of cocrystal solids, they remain susceptible to rapid dissolution of the API through the dissolution of the CCFs. However, the increasing solubility alone often does not necessarily mean an increase in permeability or bioavailability. In the study, both PROG–BBA and PROG–IPA demonstrated superior in vitro dissolution and permeation compared to PROG. However, PROG–NPA was an exception, exhibiting the highest solubility but the lowest permeability. Various factors may influence the API’s absorption: in some cases, the formulation that possess both solubilizing and inhibitory qualities (such as P-gp), can cause a simultaneous rise in permeability and solubility of API [20]. Whether the addition of BBA or IPA exerts P-gp inhibitory property or the existence of any potential factor needs further verification. For PROG–NPA, the highest solubility may be due to the interference of self-association of water molecules in the cocrystal, as water played a significant role in the network formation of PROG–NPA, thus permitting enhanced apparent solubility. However, this does not result in a significant increase in permeability, which is common in cosolvent related formulations [31]. Additionally, PROG–NPA showed a higher melting point compared with PROG–BBA and PROG–IPA, indicating there may be a possibility of stronger stability. The bioavailability of PROG–NPA might have been influenced by these factors, so it showed no significant difference compared to PROG.

## 5. Conclusions

PROG is a BCS class II drug with low solubility and high permeability, and poor water solubility is its primary challenge. In this study, a crystal engineering strategy was employed to enhance PROG solubility and bioavailability. Three novel pharmaceutical cocrystals of PROG with small-molecule acids (BBA, IPA, and NPA) were prepared and characterized using SCXRD, PXRD, FTIR, and DSC. However, the DVS experiments confirmed that the cocrystal formation did not significantly influence hygroscopicity. The solubility and dissolution behavior of PROG improved in the cocrystals. 

The *μ*Flux test of the PAMPA models indicated that the cocrystals retained the high permeability of PROG. A pharmacokinetic study in SD rats showed that the *t*_1/2_ values of all cocrystals, except for PROG–BBA, were almost twice that of free PROG. All the cocrystals increased the exposure of PROG, and the AUC_(0~∞)_ was 1–3 times that of free PROG. Notably, PROG–IPA had the highest AUC value, with approximately 3–4 times improvements in AUC_0–24 h_ and AUC_0–∞_. 

In conclusion, the cocrystal method is an effective strategy for adjusting the balance between the water solubility and bioavailability of progesterone. The hydrogen bonding modes and the water solubilities of CCFs play an important role in the process. This research may serve as a reference for developing other candidate oral drugs.

## Figures and Tables

**Table 1:** Summary of crystal structure parameters.

	PROG–BBA	PROG–IPA	PROG–NPA
Chemical formula unit	PROG·C_7_H_7_BO_3_	2PROG·C_8_H_6_O_4_·H_2_O	PROG·C_8_H_5_NO_6_·H_2_O
Empirical formula	C_28_H_37_BO_5_	C_50_H_68_O_9_	C_29_H_37_NO_9_
Formula weight	464.39	813.04	543.59
CCDC No.	2356432	2356908	2356909
Temperature/K	296 (2)	296 (2)	296 (2)
Crystal system	Orthorhombic	Monoclinic	Orthorhombic
Space group	*P*2_1_2_1_2_1_	*C*_2_	*P*2_1_2_1_2_1_
a/Å	9.2744 (17)	21.889 (6)	7.7804 (8)
b/Å	14.001 (3)	7.4735 (17)	15.6533 (15)
c/Å	19.632 (4)	16.423 (4)	22.414 (2)
α/°	90	90	90
β/°	90	125.052 (14)	90
γ/°	90	90	90
Volume/Å^3^	2549.2 (8)	2199.3 (10)	2729.8 (5)
Z	4	2	4
ρ_calc_g/cm^3^	1.215	1.228	1.323
μ/mm^−1^	0.081	0.083	0.098
F(000)	1000	880	1160
Radiation	Mo-Kα	Mo-Kα	Mo-Kα
2θ range/°	3.35 to 27.50	1.51 to 27.51	1.59 to 25.44
Index ranges	−12 ≤ h ≤ 10 −18 ≤ k ≤ 18 −25 ≤ l ≤ 21	−28 ≤ h ≤ 28 −9 ≤ k ≤ 9 −20 ≤ l ≤ 21	−8 ≤ h ≤ 9 −18 ≤ k ≤ 18 −27 ≤ l ≤ 23
Reflns collected	17,964	14,151	14,423
Unique reflns, Rint	5828, 0.0488	4693, 0.2434	5036, 0.0304
Data/restraints/parameters	5828/0/311	4693/3/275	5036/3/366
GOF	0.918	0.905	1.018
R_1_ [I > 2(I)]	0.0476	0.0675	0.0413
wR_2_(all)	0.1281	0.2764	0.1020
Largest diff. peak/hole/e Å^−3^	0.144/−0.147	0.721/−0.805	0.228/−0.234

**Table 2:** Selected hydrogen bonds in PROG cocrystals.

D–H···A	D···A (Å)	H···A (Å)	D–H···A (deg)	Symmetry Code
		PROG–BBA		
O(4)–H(4B)···O(2)	2.802 (3)	2.00	164	
O(5)–H(5B)···O(1)	2.801 (3)	1.93 (4)	168 (4)	1 − x, −1/2 + y, 1/2 − z
		PROG–IPA		
O(4)–H(4)···O(1)	2.613 (10)	1.79 (13)	160 (10)	1/2 + x, 3/2 + y, z
		PROG–NPA		
O(1W)–H(1WA)···O(6)	3.010 (3)	2.172 (16)	166 (4)	1/2 + x, −1/2 − y, −1 − z
O(1W)–H(1WB)···O(8)	2.851 (2)	2.01 (3)	172 (4)	−1/2 + x, −1/2 − y, −1 − z
O(6)–H(6)···O(1W)	2.625 (3)	1.54 (5)	174 (4)	-
O(7)–H(7)···O(1)	2.591 (3)	1.61 (3)	167 (3)	−3/2 − x, −y, −1/2 + z

## Data Availability

Data are included in the article and Supplementary Materials.

## Supplementary Materials

The following supporting information can be downloaded at: https://www.mdpi.com/article/10.3390/pharmaceutics16060816/s1, Figure S1. PXRD patterns of (A) PROG–BBA, SC, PROG, BBA; (B) PROG–IPA, SC, PROG, IPA; and (C) PROG–NPA, SC, PROG, NPA. CM: experimental pattern of cocrystal; SC: simulated PXRD pattern. Figure S2. FTIR spectra of (A) PROG–BBA, PROG, and BBA; (B) PROG–IPA, PROG, and IPA; and (C) PROG–NPA, PROG, and NPA. Figure S3. Combined DVS plots of PROG and its cocrystals. Table S1. Main pharmacokinetic parameters of free PROG and its cocrystals (mean ± SD, *n* = 4).
