# Supplementary material for: Improving the Solubility and Bioavailability of Progesterone Cocrystals with Selected Carboxylic Acids
Source: Pharmaceutics. 2024 Jun 16;16(6):816. doi: 10.3390/pharmaceutics16060816 (PMC11207217; doi:10.3390/pharmaceutics16060816)
Supplement: Supplementary file 1 [file pharmaceutics-16-00816-s001.zip › pharmaceutics-3006251-supplementary.pdf]

Supporting Information for

Improving the Solubility and Bioavailability of Progesterone Cocrystals with Selected Carboxylic Acids

Jing Xiong[2,3], Dezhong Xu[4], Hui Zhang[1], Yan Shi[5], Xiangxiang Wu[1,*], Sicen Wang[2,*]

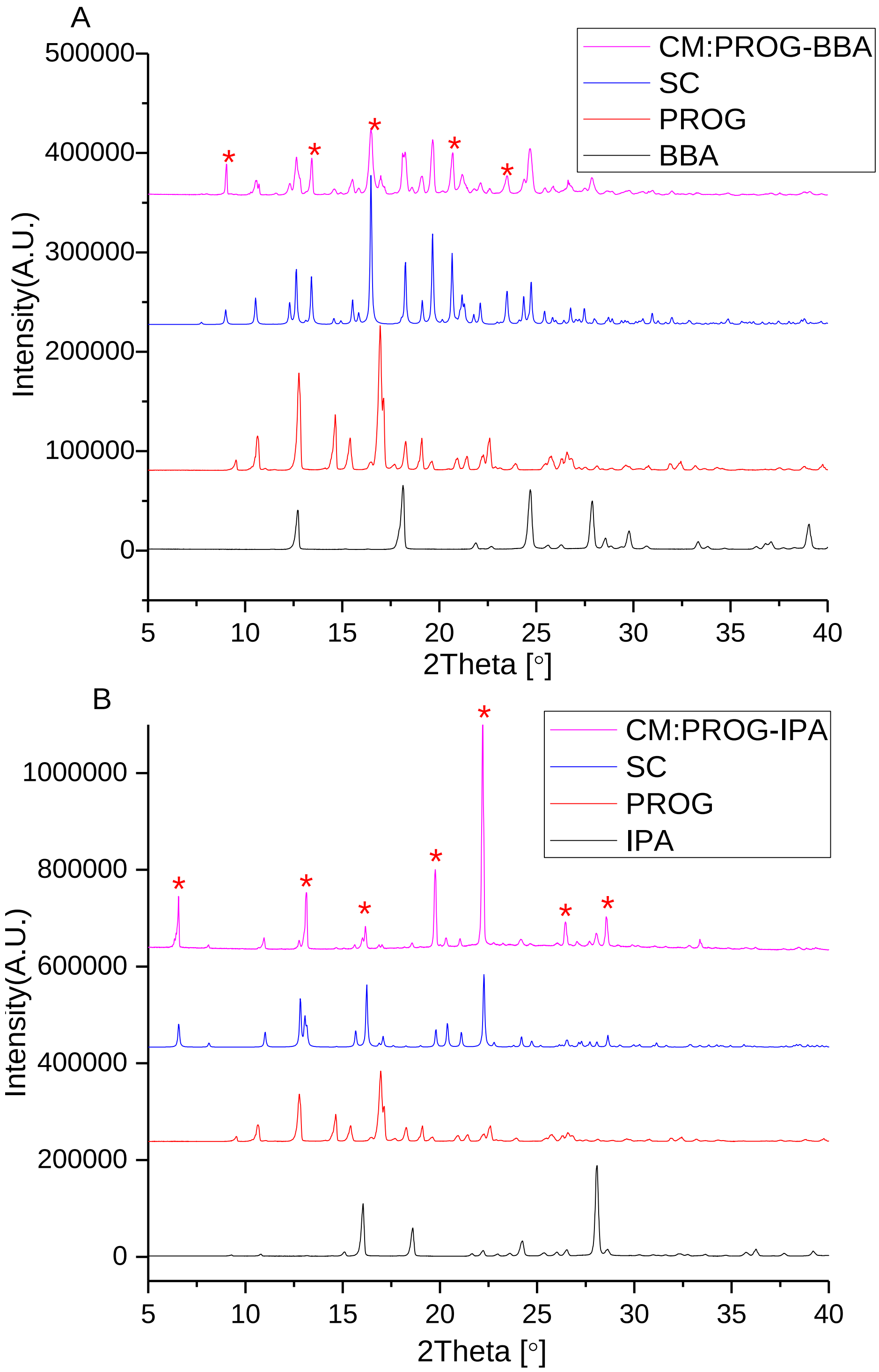
A
CM:PROG-BBA
SC
PROG
BBA
Intensity(A.U.)
2Theta [°]
B
CM:PROG-IPA
SC
PROG
IPA
Intensity(A.U.)
2Theta [°]

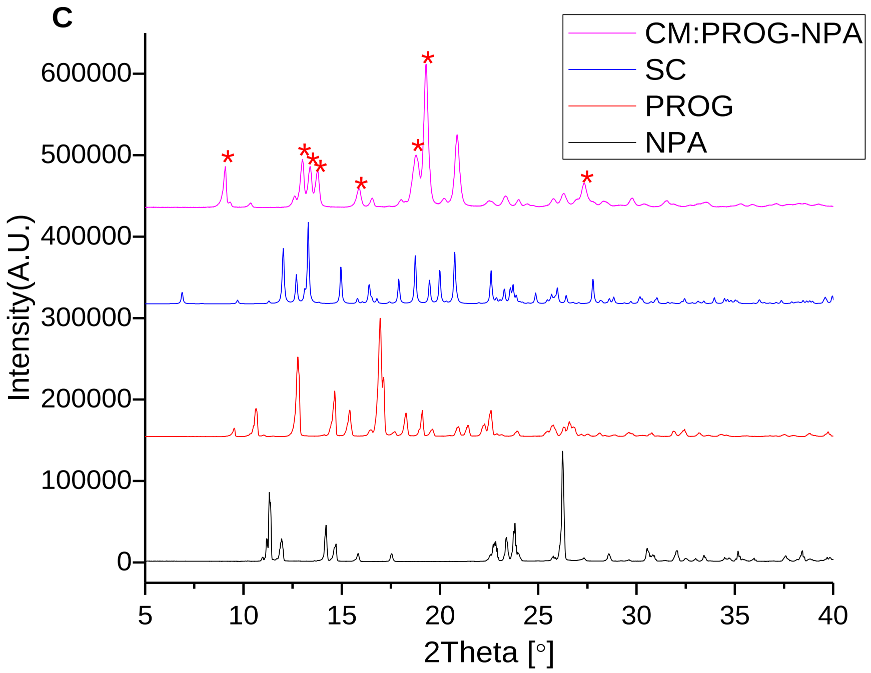

Figure S1. PXRD patterns of (A) PROG–BBA, SC, PROG, BBA; (B) PROG–IPA, SC, PROG, IPA; and (C) PROG–NPA, SC, PROG, NPA. CM: experimental pattern of cocrystal; SC: simulated PXRD pattern.

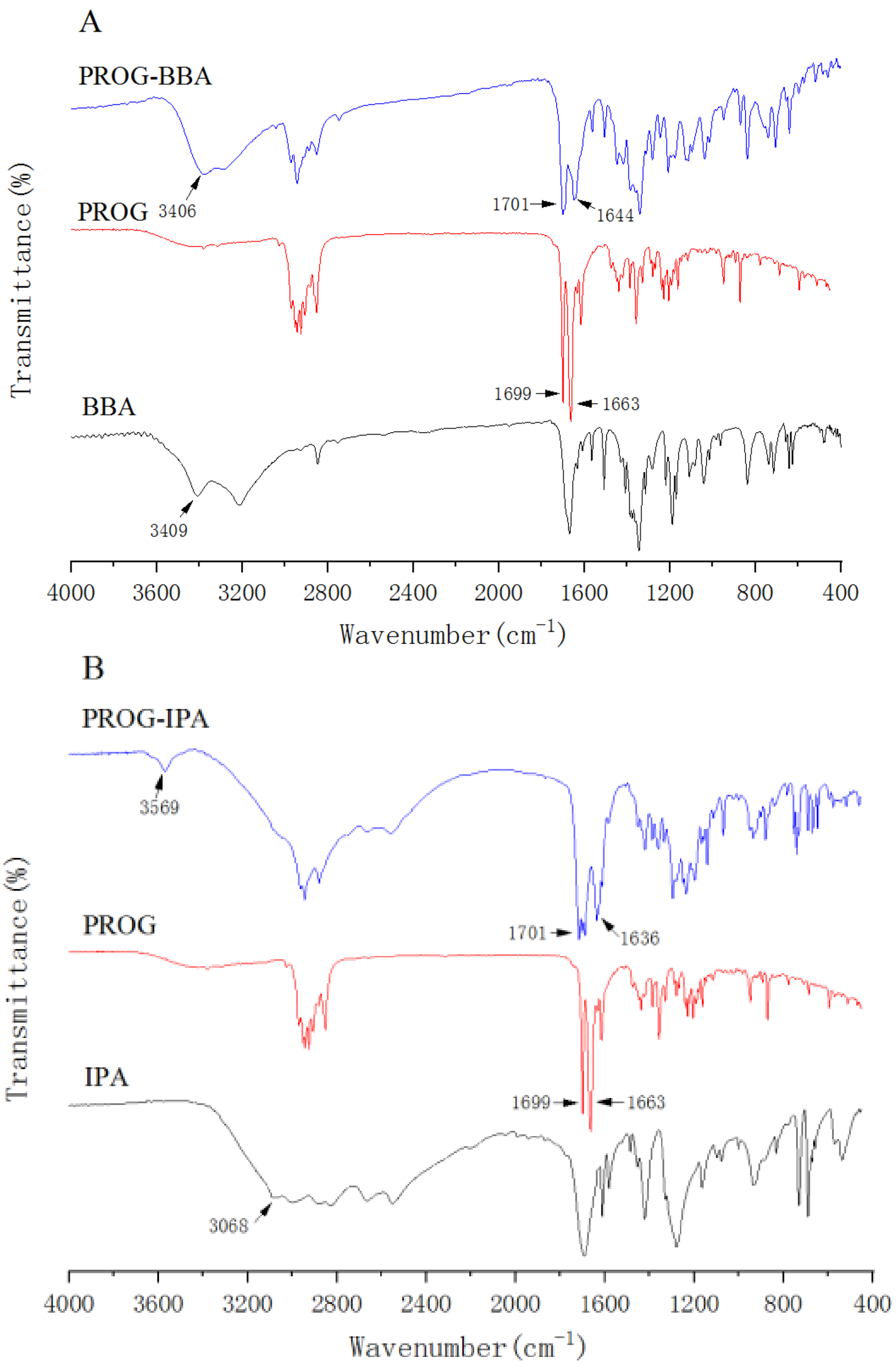
A
PROG-BBA
3406
1701
1644
PROG
1699
1663
BBA
3409
Transmittance (%)
4000
3600
3200
2800
2400
2000
1600
1200
800
400
Wavenumber (cm-1)
B
PROG-IPA
3569
1701
1636
PROG
1699
1663
IPA
3068
Transmittance (%)
4000
3600
3200
2800
2400
2000
1600
1200
800
400
Wavenumber (cm-1)

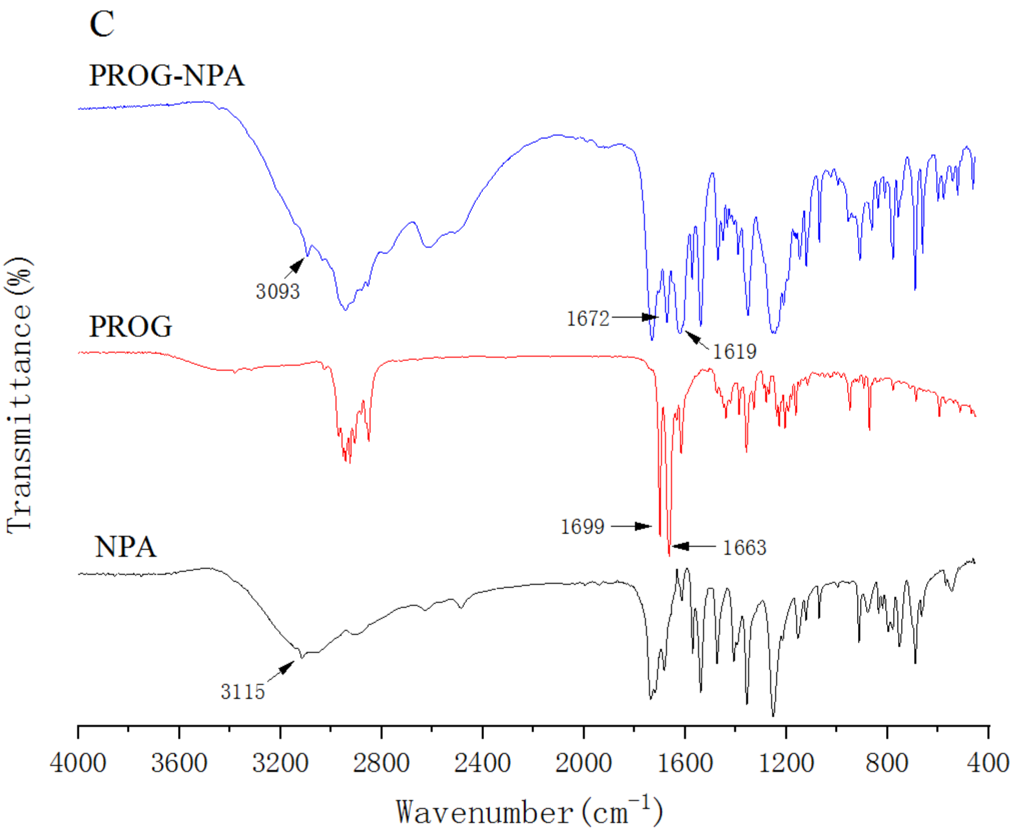

Figure S2. FTIR spectra of (A) PROG–BBA, PROG, and BBA; (B) PROG–IPA, PROG, and IPA; and (C) PROG–NPA, PROG, and NPA.

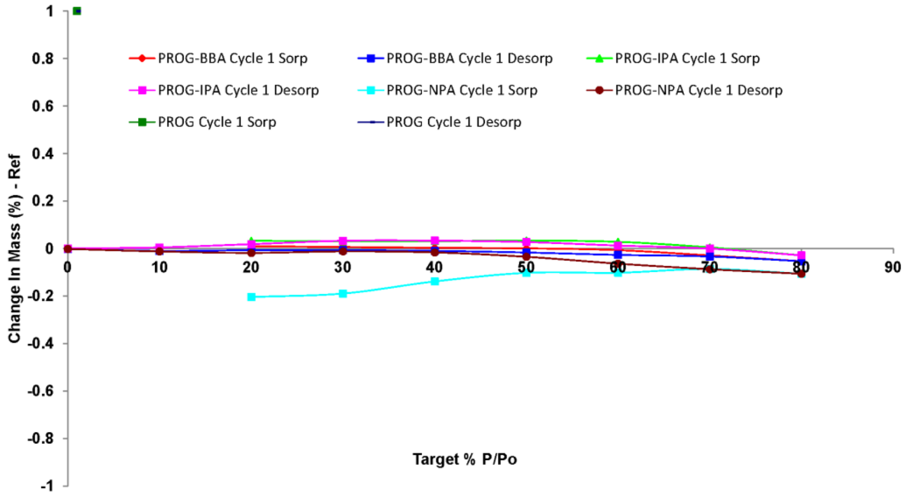

Figure S3. Combined DVS plots of PROG and its cocrystals.

Table S1. Main pharmacokinetic parameters of free PROG and its cocrystals (mean ± SD, *n* = 4).

Parameter	Unit	PROG	PROG–BBA	PROG–IPA	PROG–NPA
$t_{1/2}$	h	10.66±1.63	10.98±6.52	20.03±3.31	18.20±6.67
T_{max}	h	0.08±1.96	0.08±1.93	6.00±2.75	0.08±0.53
C_{max}	ng/mL	26.85±6.61	63.53±6.50	55.34±11.59	23.03±2.73
$AUC_{(0\sim t)}$	ng·h/mL	233.23±88.97	517.09±165.68	697.41±186.90	228.48±50.06
$AUC_{(0\sim\infty)}$	ng·h/mL	301.48±107.50	742.59±476.04	1201.72±216.77	442.67±137.58
